# Increased Incidence of Type 1 Diabetes in Children and No Change in the Age of Diagnosis and BMI-SDS at the Onset - is the Accelerator Hypothesis not Working?

**DOI:** 10.4274/jcrpe.galenos.2020.2019.0133

**Published:** 2020-09-02

**Authors:** Barbara Wasyl-Nawrot, Małgorzata Wójcik, Joanna Nazim, Jan Skupień, Jerzy B. Starzyk

**Affiliations:** 1 Hospital in Brzesko, Clinic of Pediatrics, Brzesko, Poland; 2 Department of Pediatric and Adolescent Endocrinology, Chair of Pediatrics, Pediatric Institute, Jagiellonian University, Medical College, Kraków, Poland; 3 University Children’s Hospital of Kraków, Kraków, Poland; 4 Jagiellonian University Medical College, Department of Metabolic Diseases, Kraków, Poland

**Keywords:** Accelerator hypothesis, body mass index, children, type 1 diabetes

## Abstract

**Objective:**

One of the hypothesized reasons for the observed increase in type 1 diabetes incidence in children is weight gain, causing accelerated disease development in predisposed individuals. This so-called accelerator hypothesis is, however, controversial. The aim was to analyze whether, in the ethnically homogeneous population of Lesser Poland, an increase in the number of cases of diabetes among children was associated with younger age and higher body mass index-standard deviation score (BMI-SDS) at the time of diagnosis.

**Methods:**

Retrospective data analysis from medical records of all patients <14 years (n=559; 50.6% male), with newly diagnosed type 1 diabetes, in Lesser Poland between 1^st^ January 2006 and 31^st^ December 2017 (11 years).

**Results:**

The incidence ratio ranged significantly (p<0.001) from the lowest in 2006 (11.2/100,000/year) to the highest in 2012 (21.9/100,000/year). The mean age of diagnosis was 8.2±3.5 years. There was no trend in decreasing diagnosis age (p=0.43). The mean BMI-SDS was -0.4±1.2. Almost all children (91.6%) presented with BMI-SDS within the normal range at the time of diagnosis, with only 2.7% of cases being obese and 5.7% underweight at the moment of diagnosis. There was no clear trend at all in BMI-SDS over the study period.

**Conclusion:**

These results do not corroborate an increase of type 1 incidence in paediatric population being associated with younger age of diagnosis and higher BMI-SDS. This implies that the *accelerator hypothesis* does not hold true in the study population.

**What is already known on this topic?**

An association between increase in body mass index-standard deviation score (BMI-SDS) and younger age of type 1 diabetes manifestation has been postulated. This is known as the accelerator hypothesis.

**What this study adds?**

An increase of type 1 diabetes incidence in the paediatric population of Lesser Poland was not associated with younger age of diagnosis, nor higher BMI-SDS.

## Introduction

Type 1 diabetes is one of the most common chronic diseases in children and adolescents worldwide (1). Its increasing incidence, especially in industrially developed countries, makes it necessary to look for and define potential risk factors. Some recent studies point to a possible contribution of childhood overweight and obesity to the development of type 1 diabetes at a younger age (2,3). Obesity is a well-documented risk factor for type 2 diabetes, but there are some studies indicating an association between the increase of body weight in children and adolescents and the increase in the incidence rate of type 1 diabetes in these age groups (2,3). That postulated association has been called the *accelerator hypothesis* (3,4,5). One of the arguments for the existence of such a relationship is the fact that in countries where the incidence of type 1 diabetes is increasing in the younger age groups, a simultaneous increase in the incidence of obesity in the general pediatric population was found. The number of obese children and adolescents worldwide has increased tenfold in the last 40 years, and childhood obesity, defined as equal to or greater than the 95th percentile of body mass index (BMI), has been recognized as an epidemic by the World Health Organization (6,7). The *accelerator hypothesis* identifies three processes which may accelerate the apoptosis of pancreatic beta cells: constitution, insulin resistance and autoimmunity. According to that hypothesis, weight gain causes an increase in insulin resistance which leads to a deterioration in control of blood glucose level. The rising blood glucose level accelerates beta-cell apoptosis directly via glucotoxicity, and indirectly by inducing immunogenicity (3,4,5,6,7,8). The authors of the *accelerator hypothesis* postulate that the pathogenesis of type 1 and type 2 diabetes may be, to some extent, similar and that overweight and insulin insufficiency are associated with both types of diabetes (4,5). According to the hypothesis, excessive body weight in a child who is predisposed genetically to the development of type 1 diabetes accelerates the process of beta cell destruction leading to an earlier occurrence of an overt deficit of insulin (3,6,8). This theory has been confirmed by some studies strongly supporting the association between BMI and earlier diagnosis of type 1 diabetes (6,9,10,11). Nevertheless, it is not universally accepted, as there are other studies that have contradicted these findings and do not support such a relationship (12,13,14,15). The starting point for the current study was a significant increase in the incidence of type 1 diabetes in the young age groups in the Lesser Poland region (from 5.2/100,000/year in 1987 to 21.9/100,000/year in 2012), which was demonstrated in our previous paper (16). Simultaneously, there was evidence of an increase in obesity incidence in the general paediatric population over the same time period and in the same geographic area (17).

## Objective

The aim of the study was to analyze whether, in the ethnically homogeneous population of Lesser Poland, an increase in the number of cases of diabetes among children was associated with younger age and higher BMI-standard deviation (SD) score (SDS) at the time of diagnosis.

## Methods

Retrospective data was extracted from medical records of all patients under the age of 14 years, with newly diagnosed type 1 diabetes in the Krakow region (former *województwo krakowskie*) between 1^st^ of January 2006 and 31^st^ of December 2012 and in the whole of Lesser Poland between 1^s^t of January 2013 and 31^st^ of December 2017 and analyzed. The analysis included children with type 1 diabetes only; patients with other types of diabetes were excluded. Type 1 diabetes was defined as acute-onset diabetes presenting with ketoacidosis and/or symptoms of polyuria, polydipsia and weight loss, complete insulin dependence within <1 year from diagnosis, or positive anti-glutamic acid decarboxylase or anti-IA2 test on diabetes diagnosis. All data were collected in one centre for the region; the Department of Pediatric and Adolescents Endocrinology, Chair of Pediatrics, Jagiellonian University Medical College, which is the reference centre for the region.

Body weight and height were measured to the nearest 0.1 kg and 0.1 cm, respectively, using a stadiometer (Harpenden, UK) and a balanced scale (Seca 700, Germany). All measurements were done during the hospitalization at the moment of diabetes diagnosis, after normalization of the general condition and rehydration. As the standard of reference for calculating BMI-SDS, normal values from the local population were used (18). Incidence rate was evaluated based on the data from the Central Statistical Office (Polish: Glowny Urzad Statystyczny) for the population of the region which was subject to analysis (19).

### Statistical Analysis

Statistics data are presented as means with SD or medians and quartiles for continuous variables, or counts and percentages for categorical variables. Univariate and multiple regression models were used to test the association between BMI-SDS, age of diagnosis and calendar year of diagnosis. P values <0.05 were considered significant. All calculations were performed using the Microsoft Excel and SAS 9.3 software.

### Ethics

The study was conducted in accordance with the requirements of ethics, with particular regard to the protection of sensitive data. No additional consent from the bioethics committee was required due to the retrospective nature of the studies. Parents (legal guardians) and study participants gave informed consent for the later use of anonymised data.

## Results

**Figure 1:**
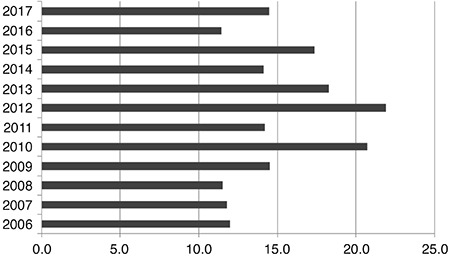
Incidence rates (per 100,000) for type 1 diabetes in Krakow and Lesser Poland region (Krakow only up to 2012 then figures represent the whole of Lesser Poland)

**Figure 2:**
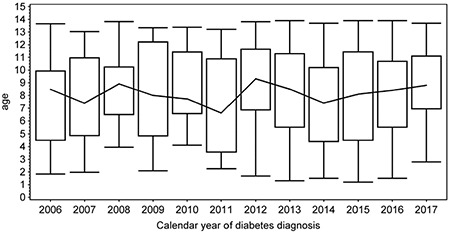
Box plot of mean age at the time of type 1 diabetes diagnosis in sequencially analyzed calendar years. The upper and lower box boundaries indicate quartiles and the line across the centre of the graph joins the medians. The whiskers indicate the range of age in each year

**Figure 3:**
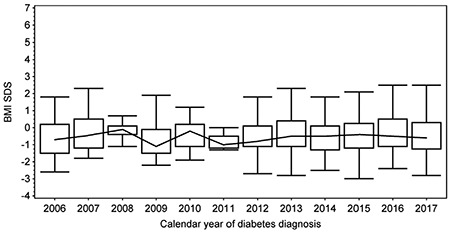
Box plot of mean age at the time of type 1 diabetes diagnosis in sequencially analyzed calendar years. The upper and lower box boundaries indicate quartiles and the line across the centre of the graph joins the medians. The whiskers indicate the range of age in each year BMI-SDS: Body mass index-standard deviation score

**Figure 4:**
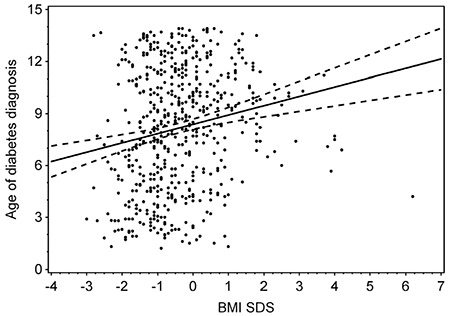
Box plot of mean body mass index-standard deviation score (BMI-SDS) at the time of type 1 diabetes diagnosis in sequencially analyzed calendar years. The upper and lower box boundaries indicate quartiles and the line across the centre of the graph joins the medians. The whiskers indicate the range of BMI-SDS in each year

There were 559 (50.6% male) cases of type 1 diabetes diagnosed before age 14. The incidence ratio ranged significantly (p<0.001) from the lowest in 2006 (11.2/100,000/year) to the highest in 2012 (21.9/100,000/year) (Figure 1). The median (interquartile range) age of diagnosis was 8.4 (5.4 to 11.1) years, and the mean age was 8.2±3.5 years. The mean BMI-SDS was -0.4±1.2, indicating type 1 diabetes onset in individuals somewhat leaner than the reference population. This reflects the weight loss characterizing the onset of diabetes. During the study period, there was no trend of decreasing age at the time of diagnosis (Figure 2, p=0.43). Almost all children (91.6%) presented with BMI-SDS within the normal range at the time of diagnosis, with only 16 (2.7%) of cases being obese and 5.7% underweight at the moment of diagnosis (Table 1). There was no association between calendar year of diabetes diagnosis and BMI-SDS (p=0.87, see Figure 3). A significant relationship was observed between the age of diabetes diagnosis and BMI-SDS. An increase in BMI-SDS by 1 SD was associated with the development of the disease 0.54 years of age later (Figure 4) and this association remained unchanged after adjusting for sex and calendar year of diabetes diagnosis (p<0.001). This association is the reverse of what would be expected from the accelerator hypothesis.

## Discussion

The incidence of type 1 diabetes, as well as overweight and obesity in children are increasing in Poland (13,17). The present study investigates whether these phenomena are interrelated or only co-exist in the same time and place. This continuous and longitudinal study is the first such study conducted in our country. According to the current definitions, type 1 diabetes mellitus is an autoimmune chronic disease in children and adolescents determined by insulin insufficiency, while type 2 diabetes mellitus is a metabolic disorder in adults and the elderly population, associated with obesity and insulin resistance (13). The *accelerator hypothesis* attempts to unify both types of diabetes as the same insulin secretion disorder, but with a different background (3,6,8). The rate of beta cell damage and insulin loss in type 1 diabetes seems to be associated with more susceptible genotypes and the influence of undefined environmental factors. To date there are more than 60 genomic loci identified, but human leukocyte antigen 6p21 has the strongest association with type 1 diabetes development, and islet autoimmunity develops in about 5% of people with that genetic predisposition (19,20). Contrary to the well-defined genetic background, most of the environmental factors contributing to beta cell loss remain unidentified (21). One of the problems that has been investigated is the possible impact of overweight on the acceleration of insulin insufficiency. In recent years, many trials have been conducted to prove this theory. Studies conducted in different ethnic settings worldwide reported conflicting results (inverse, positive or lack of correlation between body weight and the development of type 1 diabetes). A Norwegian cohort study pointed to an increased risk of autoimmunity in individuals with high-risk genotype with a weight gain of over 15 kg within the first year of life and/or maternal BMI during pregnancy over 30 kg/m^2^ (19). Even stronger evidence supporting the *accelerator hypothesis* was provided by the Southeastern Wisconsin study that revealed a significant inverse correlation between BMI and age of diagnosis (9). In a European study, Knerr et al (10) also demonstrated that elevated BMI has an impact on younger age of diabetes onset. Slightly more cautious conclusions were drawn from the study by Dabelea et al (11) that indicated an inverse correlation but only in children with already reduced beta cell function indicated by fasting C-peptide level below the median. In our study, we did not confirm an association between higher BMI-SDS and age at the moment of diagnosis. Indeed, in younger children there was a small association in the opposite direction. Similar observations were also made by authors investigating this phenomenon in various parts of the world. Over 20 years of observation of Australian children under 16 years of age with type 1 diabetes, Islam et al (13) reported that the number of overweight and obese children has remained relatively stable, despite an increase in the incidence rate of type 1 diabetes. Moreover, Derraik et al (12) showed that the mean BMI-SDS of newly diagnosed type 1 diabetes over the period 1990-2009 in New Zealand did not alter in comparison to the general population. Also in our group the incidence of obesity at the moment of type 1 diabetes diagnosis was comparable to the general population living in the same geographic area (17). The prevalence of overweight and obesity is not regulary screened among Polish children. There is no national registry, therefore the precise, reliable data is not available. Nevertheless, in recent years several studies have been published on the prevalence of overweight and obesity in children and adolescents living in different regions of Poland. However, mainly due to differences in research methodology, their results are quite divergent. For example, in Gdańsk, in the North of Poland, obesity was found in 1.5-7.5% of subjects, depending on sex and age at the time of the study (22). In a study conducted among seven-year-olds in a city of Wrocław, in Lower Silesia, South-Western Poland, obesity was found to vary between 10.7-26.6% of children, depending on the place of residence (23). In South-Eastern Poland in 2012, the prevalence of obesity in preschool children was 10.8% (24). In the region covered by our study, the prevalence of obesity in adolescents aged between 14-18 years old is 4.2% (17). To some extent the prevalence of excess weight may be obscured by the fact that anthropometric measurement were taken at diabetes diagnosis. The effect of dehydration may be ruled out, but some degree of loss of body mass has persisted at that time. That is an undoubted limitation of this study, as well as other similar papers. All the weight measurement took place after the diagnosis of the disease on the basis of clinical symptoms. Although the body weight values analyzed came from measurements made after rehydration, resolution of acidosis and improvement of general condition, they cannot take into account the full weight loss associated with lypolysis of adipose tissue before the onset of diabetes. In fact, such an analysis is not possible, because in the first phase type 1 diabetes occurs without clinically overt symptoms and it is not possible to accurately determine its onset, and thus accurate determination of BMI-SDS at the time of actual onset of the disease is also not possible. We were unable to use body weight from before diabetes onset, as regular and standardized measurement were never taken on regular basis in healthy children. Since weight loss would affect all study subjects, we do not expect that this issue has altered the relationships between age of diabetes diagnosis and BMI-SDS. Recently published data from over 360,000 British children and young adults (<25 years old) observed during 1994-2013 did not show any significant correlation between BMI and type 1 diabetes incidence. Interestingly, the authors pointed to a slightly higher incidence rate in overweight, but not in obese children (14). Attempts to include additional ethnic factors showed that the *accelerator hypothesis* is not universal (15). Interestingly, we found that BMI-SDS was significantly higher in the older age groups. An increase in BMI-SDS by 1 SD was associated with the development of the disease 0.54 years of age later. This novel observation seems to be particularly important if we take into consideration the possible impact of puberty on insulin secretion. The relationship between obesity, puberty and type 2 diabetes incidence is clear and well documented (25). During puberty, growth hormone and cortical secretion increases, causing physiological insulin resistance (2). Therefore type 2 diabetes almost never occurs in children before puberty. Our results point to a potential contribution of increased body weight during puberty on the age of type 1 diabetes manifestation. Perhaps, the *accelerator hypothesis* may therefore partly explain the incidence of type 1 diabetes in the period of puberty. However, this would be very difficult to prove but warrants further investigation.

### Study Limitations

The main limitation of our study is the inclusion of a relatively small group of participants. However, taking into consideration that we analyzed all new cases of type 1 diabetes under the age of 14 in the homogeneous Lesser Poland population, the results can be considered valuable. To obtain more reliable data, it would be advisable to perform a similar analysis for a national cohort or, optimally, a multinational study.

## Conclusion

These results do not corroborate an increase of type 1 incidence in a paediatric population being associated with younger age of diagnosis and higher BMI-SDS. This implies that the *accelerator hypothesis* does not hold true in the studied population.

## Figures and Tables

**Table 1:**
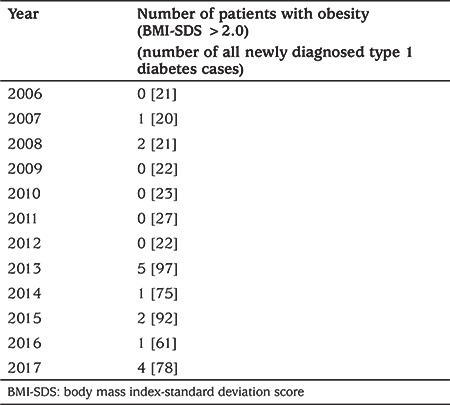
The occurrence of obesity (body mass index-standard deviation score >2.0) in patients with newly diagnosed type 1 diabetes in sequencial years of observation
